# *Mycobacterium kyorinense* Infection 

**DOI:** 10.3201/eid1903.12-0591

**Published:** 2013-03

**Authors:** Hiroaki Ohnishi, Shota Yonetani, Satsuki Matsushima, Hiroo Wada, Kei Takeshita, Daisuke Kuramochi, Paulo Cesar de Souza Caldas, Carlos Eduardo Dias Campos, Bianca Porphirio da Costa, Jesus Pais Ramos, Shinichirou Mikura, Eriko Narisawa, Akira Fujita, Yasunori Funayama, Yoshihiro Kobashi, Yumi Sakakibara, Yukako Ishiyama, Shunji Takakura, Hajime Goto, Takashi Watanabe

**Affiliations:** Author affiliations: Kyorin University School of Medicine, Tokyo, Japan (H. Ohnishi, S. Yonetani, S. Matsushima, H. Wada, H. Goto, Y. Ishayama, T. Watanabe);; Kitasato University–Kitasato Institute Hospital, Tokyo (K. Takeshita);; International University of Health and Welfare Hospital, Tochigi, Japan (D. Kuramochi);; Centro de Referência Prof. Helio Fraga, Rio De Janeiro, Brazil (P.C.d.S Caldas, C.E.D. Campos, B.P. da Costa, J.P. Ramos); Tokyo Metropolitan Tama Medical Center, Tokyo (S. Mikura, E. Narisawa, A. Fujita);; Tsukuba Gakuen Hospital, Ibaraki, Japan (Y. Funayama); Kawasaki Medical School, Okayama, Japan (Y. Kobashi); Toshiba Hospital, Tokyo (Y. Sakakibara);; Kyoto University Graduate School of Medicine, Kyoto, Japan (S. Takakura)

**Keywords:** pneumonia, lymphadenitis, arthritis, *Mycobacterium celatum*, *rpoB*, rifampin, mutation, bacteria

**To the Editor:** *Mycobacterium kyorinense* is a nonpigmented, slowly growing mycobacterium that was initially isolated in 2007 from a patient with pneumonia in Japan (*1**,**2*). The sequences of the 16S rRNA, *hsp65*, and *rpoB* genes of *M. kyorinense* were closely related to, but different from, those of the type strains of *M. celatum* and *M. branderi*, the 2 most phylogenetically related species (*1*). Biochemical tests, such as those for arylsulfatase activity, tellurite reduction, and heat-stable catalase, also distinguish *M. kyorinense* from *M. celatum* and *M. branderi*. In our initial report, in which this species was first recognized, we described 3 strains isolated from Japanese patients (*1*). Recently, 1 additional case was reported in Brazil (*3*). Here we describe 7 newly identified patients whose infection may have been caused by *M. kyorinense*.

In reviewing the characteristics of these 11 patients (10 from Japan and 1 from Brazil), we found no apparent contacts among them. Nine of the 11 patients had respiratory infections, 1 had lymphadenitis, and 1 had arthritis (Table). Of these, 9 patients fulfilled the criteria for infections of clinical significance (*4*) and were considered to harbor infection by *M. kyorinense*. Of the 9 patients with respiratory infections, 4 died as a result of the infection. These data suggest that *M. kyorinense* belongs to a class of nontuberculous mycobacteria that are pathogenic for humans and have substantial clinical effects.

**Table:** Clinical characteristics of patients infected with *Mycobacterium kyorinense* and antimicrobial susceptibility of the organism*

Characteristic	Patient/strain no.										
	1†	2‡	3‡	4	5	6	7	8	9	10	11§
Year of diagnosis	2005	2006	2008	2008	2009	2009	2010	2010	2011	2011	2007
Age, y	89	64	70	81	50	67	72	48	66	60	26
Sex	M	F	M	M	M	M	M	F	M	M	M
Major infection site	Lung	Lung	Lung	Lung	Lymph node	Lung	Lung	Joint	Lung	Lung	Lung
Specimen	Sputum	Sputum	Sputum	BAL	Pus	BAL	Sputum	Pus	Sputum	Sputum	Sputum
Coexisting disease	COPD	Breast cancer	None	None	MDS	None	None	RA, SLE	None	COPD	NA
Country	Japan	Japan	Japan	Japan	Japan	Japan	Japan	Japan	Japan	Japan	Brazil
AM drug MIC, μg/mL											
STR	0.25	0.25	0.25	S	ND	0.25	0.125	0.5	0.25	0.5	S
EMB	4	4	2	R	128	2	1	2	4	4	S
KAN	0.5	1	0.25	S	ND	0.5	0.5	0.5	0.5	0.5	ND
INH	16	16	32	R	2	0.5	8	4	1	4	R
RIF	32	32	32	R	32	32	32	32	32	32	R
LVX	0.125	0.125	0.03	R	0.25	0.06	0.06	0.125	0.125	0.25	ND
CLR	0.03	0.03	0.03	ND	0.03	0.03	ND	ND	0.03	0.125	ND
AMK	0.5	0.5	0.5	ND	0.5	1	ND	0.5	0.5	0.5	S
Clinical efficacy of AM drug combination											
Efficacious	None	None	None	None	CLR, RIF, LVX, AMK	CLR, STR, MXF	None	LVX, EMB, CLR	RIF, CLR, LVX	CLR, LVX	NA
Nonefficacious	BIP	None	RFB, EMB, CLR	INH, EMB, RIF	None	RIF, EMB, CLR, RIF, AZM, LVX	CLR, RIF, EMB	INH, RIF, EMB	INH, RIF, EMB	RFB, EMB	NA
Outcome	Dead	Dead	Dead	Dead	Alive	Alive	Alive	Alive	Alive	Alive	Dead

*BAL, bronchoalveolar lavage; COPD, chronic obstructive pulmonary disease; MDS, myelodysplastic syndrome; RA, rheumatoid arthritis; SLE, systemic lupus erythematosus; NA, not available; AM, antimicrobial; STR, streptomycin; S, sensitive; ND, not done; EMB, ethambutol; R, resistant; KAN, kanamycin; INH, isoniazid; RIF, rifampin; LVX, levofloxacin; CLR, clarithromycin; AMK, amikacin; AM, antimicrobial; RFB, rifabutin; MXF, moxifloxacin; BIP, biapenem; AZM, azithromycin.  †Reported in (*1,2*).  ‡Reported in (*1*). §Reported in (*3*). ¶Strains 1–10 (except for 4): assayed by broth microdilution MIC for nontuberculosis mycobacteria (BrothMIC NTM; Kyokuto Pharmaceutical Industrial Co., Ltd., Tokyo, Japan). Strains 4 and 11: susceptibility test performed by using Vite Spectrum SR (Kyokuto Pharmaceutical Industrial Co., Ltd.) and BACTEC MGIT 960 Mycobacterial Detection System (Becton, Dickinson and Company, Franklin Lakes, NJ, USA), respectively; therefore, numeric MIC data were not available for these strains.

Among the 10 patients for whom precise clinical records were available, 7 patients were treated with first-line tuberculosis drugs, mainly rifampin, isoniazid, and ethambutol, but these therapies were ineffective for all patients. Six patients received a combination of antimicrobial drugs, including macrolides and fluoroquinolones, as first- or second-line chemotherapy, and infection was subdued without recurrence in 5 patients. In contrast, 4 patients with pneumonia who did not receive sufficient therapy with the latter regimen eventually died of infection (3 patients) or breast cancer (1 patient).

MICs of various antimicrobial drugs for the 9 strains of *M. kyorinense* were determined by the broth microdilution method as described (*1*). For most strains, the MICs of rifampin, ethambutol, and isoniazid were relatively high, and MICs of macrolides, aminoglycosides, and quinolones were relatively low. Notably, MICs of rifampin were remarkably high (>32 μg/mL) for all tested strains (Table).

Direct sequencing of the 16S rRNA gene, performed as previously described, revealed that 8 of the 9 available *M. kyorinense* isolates were identical across the entire sequenced interval (1,470 bp). The sole exception was the strain from Brazil, which showed a 4-bp substitution that the other strains did not (*3*). Although the other 8 strains had identical 16S rRNA sequences, all showed heterogeneity at 9 positions that had not been observed to be heterogeneous in the previous investigation (*1*). This observation might reflect the presence of 2 copies of the 16S rRNA gene, as has been occasionally reported for other mycobacterial species, including *M. celatum* (*5*). Direct sequencing of the entire *rpoB* gene demonstrated that all strains had identical sequences for this locus. The strains differed from the sequence of *M. tuberculosis* at 15 nt within codons 511–533. At the amino acid level, these changes were synonymous for the 2 species, with the exception of amino acid residue 531. This residue, Ser531 in the *M. tuberculosis* RpoB protein, was replaced by an Asp in *M. kyorinense*. Notably, Ser531 is the most frequent location of substitutions in rifampin-resistant strains of *M. tuberculosis* (*6*).

Why *M. kyorinense* has been isolated almost exclusively in Japan is not clear. This tendency may be largely caused by a reporting bias in Japan. However, *M. kyorinense* may have a particular geographic distribution. In this context, it is noteworthy that the sole strain from Brazil characterized in the current study differed slightly in 16S rRNA sequences from the strains isolated in Japan.

It also is notable that the *M. kyorinense* strains isolated so far were invariably resistant to rifampin by in vitro susceptibility testing. Rifampin appeared to have been clinically ineffective in most patients, although definite efficacy of antimicrobial drugs cannot be evaluated by this retrospective type of study. Analysis of the *rpoB* gene sequence of *M. kyorinense* revealed the replacement of aa 531 when compared to the *rpoB* gene sequence of the *M. tuberculosis* protein. This finding suggests that *M. kyorinense* is inherently resistant to rifampin because of the structural features of its RpoB protein. Amino acid replacement at RpoB residue 531 also has been reported in other bacterial species resistant to rifampin, such as *M. celatum*, *Borrelia burgdorferi,* and *Spiroplasma citri* (*7*–*9*). In any case, understanding the intrinsic resistance of *M. kyorinense* to rifampin is critical for appropriately treating infection by this microorganism. On the basis of the results of our study, we recommend that a combination of fluoroquinolones and macrolides and/or aminoglycosides be used for the initial treatment of infection by *M. kyorinense* in most patients. 
